# Validation of prediction models of severe disease course and non-achievement of remission in juvenile idiopathic arthritis: part 1—results of the Canadian model in the Nordic cohort

**DOI:** 10.1186/s13075-019-2060-2

**Published:** 2019-12-05

**Authors:** Veronika Rypdal, Jaime Guzman, Andrew Henrey, Thomas Loughin, Mia Glerup, Ellen Dalen Arnstad, Kristiina Aalto, Marite Rygg, Susan Nielsen, Troels Herlin, Anders Fasth, Lillemor Berntson, Martin Rypdal, Ellen Nordal, for the ReACCh-Out and NoSPeR Investigators

**Affiliations:** 1 Department of Pediatrics, University Hospital of North Norway, Tromsø, Norway; 2 Department of Clinical Medicine, UiT – The Arctic University of Norway, Tromsø, Norway; 3 Department of Pediatrics, BC Children’s Hospital and University of British Columbia, Vancouver, British Columbia Canada; 4 Department of Statistics and Actuarial Sciences, Simon Fraser University, Burnaby, British Columbia Canada; 5 Department of Pediatrics, Aarhus University Hospital, Aarhus, Denmark; 6 Department of Clinical and Molecular Medicine, NTNU - Norwegian University of Science and Technology, Trondheim, Norway; 7 Department of Pediatrics, Levanger Hospital, Nord-Trøndelag Hospital Trust, Levanger, Norway; 8 Department of Pediatrics, Helsinki University Hospital, University of Helsinki, Helsinki, Finland; 9 Department of Pediatrics, St. Olavs Hospital, Trondheim, Norway; 10 Department of Pediatrics, Rigshospitalet Copenhagen University Hospital, Copenhagen, Denmark; 11 Department of Pediatrics, Institute of Clinical Sciences, Sahlgrenska Academy, University of Gothenburg, Gothenburg, Sweden; 12 Department of Women’s and Children’s Health, Uppsala University, Uppsala, Sweden; 13 Department of Mathematics and Statistics, UIT – The Arctic University of Norway, Tromsø, Norway

**Keywords:** Juvenile idiopathic arthritis, Prediction, Validation, Outcome research, Remission

## Abstract

**Background:**

Models to predict disease course and long-term outcome based on clinical characteristics at disease onset may guide early treatment strategies in juvenile idiopathic arthritis (JIA). Before a prediction model can be recommended for use in clinical practice, it needs to be validated in a different cohort than the one used for building the model. The aim of the current study was to validate the predictive performance of the Canadian prediction model developed by Guzman et al. and the Nordic model derived from Rypdal et al. to predict severe disease course and non-achievement of remission in Nordic patients with JIA.

**Methods:**

The Canadian and Nordic multivariable logistic regression models were evaluated in the Nordic JIA cohort for prediction of non-achievement of remission, and the data-driven outcome denoted severe disease course. A total of 440 patients in the Nordic cohort with a baseline visit and an 8-year visit were included. The Canadian prediction model was first externally validated exactly as published. Both the Nordic and Canadian models were subsequently evaluated with repeated fine-tuning of model coefficients in training sets and testing in disjoint validation sets. The predictive performances of the models were assessed with receiver operating characteristic curves and C-indices. A model with a C-index above 0.7 was considered useful for clinical prediction.

**Results:**

The Canadian prediction model had excellent predictive ability and was comparable in performance to the Nordic model in predicting severe disease course in the Nordic JIA cohort. The Canadian model yielded a C-index of 0.85 (IQR 0.83–0.87) for prediction of severe disease course and a C-index of 0.66 (0.63–0.68) for prediction of non-achievement of remission when applied directly. The median C-indices after fine-tuning were 0.85 (0.80–0.89) and 0.69 (0.65–0.73), respectively. Internal validation of the Nordic model for prediction of severe disease course resulted in a median C-index of 0.90 (0.86–0.92).

**Conclusions:**

External validation of the Canadian model and internal validation of the Nordic model with severe disease course as outcome confirm their predictive abilities. Our findings suggest that predicting long-term remission is more challenging than predicting severe disease course.

## Background

Population-based studies show that juvenile idiopathic arthritis (JIA) is a chronic childhood rheumatic disease with diverse disease manifestations, courses, and prognoses [1–4]. Prognostic prediction models are increasingly important tools for informed decision-making in medicine [5, 6]. In a newly diagnosed patient with JIA, it can be challenging to decide if a potent treatment with possible serious side effects should be started early in the disease course. A well-performing prediction model can help assess the risk of severe disease and hence guide decisions on starting or stepping up disease-modifying antirheumatic drugs, including biologic treatments. This may facilitate individually tailored treatment strategies within the so-called window of opportunity [7–10]. Before such prediction models can be recommended for general use in clinical practice, we need to ensure they have good predictive performance across different JIA populations. Unfortunately, studies on development of prediction models in pediatrics [11] and in JIA are scarce [12–18]. As far as we know, no study has previously reported a quantitative external validation of prediction models in JIA in a different population.

To address this knowledge gap, a collaboration has been initiated between two prospective and well-defined longitudinal cohort studies: the Research in Arthritis in Canadian Children Emphasizing Outcomes (ReACCh-Out) Cohort and the Nordic JIA cohort. The first results of the collaboration are presented here and in the twin study by Henrey et al. (part 2). These studies analyze prediction models recently proposed by Guzman et al. [17] and Rypdal et al. [19]. Guzman et al. constructed a model for predicting *severe disease course* derived from the ReACCh-Out study (the Canadian model). The model had a C-index of 0.85 in internal validation in the Canadian cohort. Rypdal et al. constructed a model for prediction of *non-achievement of remission* (the Nordic model), and this model had a C-index of 0.78 in internal validation in the Nordic cohort.

In the present study, our aims were to validate the predictive ability of the Canadian model in the Nordic JIA cohort and to internally validate the performance of the Nordic model to predict *severe disease course*, an outcome originally constructed from data in the Canadian cohort [17, 20]. Conversely, the Nordic prediction model was tested for these outcomes in the Canadian cohort, with results presented in the twin paper by Henrey et al. (part 2). The validated prediction models may in the future be updated, harmonized, and eventually used as clinical tools in decision-making regarding early individualized treatment in JIA.

## Patients and methods

The Nordic JIA study is a prospective, longitudinal, multicenter cohort [2, 21]. Measures were taken to ensure a population-based approach; all consecutive newly diagnosed JIA patients from 12 pediatric rheumatology centers in defined geographical areas of Denmark, Finland, Norway, and Sweden were included if disease onset was between January 1, 1997, and June 30, 2000, and the International League of Associations for Rheumatology criteria for JIA [2] were fulfilled. The aim was to have a baseline visit 6 months after disease onset, and the patients were followed at regular visits with 1-to-3-year intervals up to 8-years after disease onset.

The ReACCh-Out study is also a multicenter prospective study. A total of 16 pediatric rheumatology centers across Canada participated, and consecutive patients with newly diagnosed JIA were recruited between January 2005 and December 2010. The first visit occurred as soon as possible after diagnosis, but the time from diagnosis to the first visit could be up to 1 year. The inclusion criterion in the Canadian prediction study was attendance in at least 6 of 8 study visits, which were scheduled every 6 months for 2 years, and then yearly up to 5 years. It was also required that information was available at least at one visit, for each of the 5 clinical variables used to construct the *severe disease course* outcome [17].

Both studies collected extensive clinical and laboratory data at the study visits as previously reported [17, 19]. Characteristics of the two study populations are presented in Table 1.

**Table 1:** JIA-study population in the Canadian ReACCh-Out and the Nordic JIA cohort

Characteristics	Canadian development cohort	Nordic validation cohort
Study design	Prospective multicenter	Prospective multicenter
Patient recruitment period^a^	January 2005–December 2010	January 1997–June 2000
Total participants, *n*	1497	500
Time from onset to baseline study visit, months^b^	5.8 (3.0–11.0)	7.0 (6.0–8.0)
Time from onset to outcome assessment, months^b^	49 (38–59)^c^	98 (95–102)
Participants in the current study, *n*	609	440
Inclusion criteria	6 of 8 study visits^d^	Baseline and 8-year study visit
Main outcome	Severe disease course	Non-achievement of remission

^a^Newly diagnosed JIA patients

^b^Median interquartile range (IQR)

^c^Severe disease course outcome was assessed over time, not at a single point

^d^Additionally, at least one value available for each of the five patient-relevant variables

The current study is reported according to the TRIPOD guideline (Transparent reporting of a multivariable prediction model for individual prognosis or diagnosis) [4, 22].

### Patients

The present study includes all patients from the Nordic cohort with data available from at least a baseline and an 8-year visit. This includes 440 (88%) of the 500 patients originally included at baseline. In contrast to the previous work on prediction models in the Nordic cohort [19], patients with systemic JIA are included in the current study.

### Outcomes

The main outcome predicted in the previous Nordic study was *non-achievement of remission* at the 8-year visit, which included patients with active disease, inactive disease on medications, or inactive disease off medications for less than 12 months. Inactive disease was defined by the Wallace 2004 criteria, the current criteria at the time the 8-year study was conducted [23, 24].

The main outcome in the Canadian study was *severe disease course*. The method used to develop and define this outcome was previously reported [17]. In summary, the clinical JIA course was described according to five variables: participant-defined quality of life and pain reports, both assessed on 10-cm visual analogue scales (VAS); active joint count; medication requirements; and medication side effects. Based on this information, four different clinical courses were identified by a clustering algorithm. The main outcome, *severe disease course*, was the union of the two worst groups, severe controlled course and severe persistent course, as defined by Guzman et al. [17].

In the present study, a version of the Canadian outcome was constructed in the Nordic cohort using information on four variables collected at the 8-year study visit. This outcome is also denoted *severe disease course*, but the construct variables in the Nordic cohort were the cumulative active joint count, the remission status, the Childhood Health Assessment Questionnaire disability index (CHAQ), and the Physical Summary Score (PhS) derived from the Child Health Questionnaire Parent form (CHQ-PF50) [25]. The aim was to construct a *severe disease course* group corresponding as closely as possible to the outcome used in the ReACCh-Out prediction study. Accordingly, we used these four variables and a clustering algorithm to divide the Nordic cohort in four disease course groups. The two most severe courses were defined to have a *severe disease course*. Characteristics of the four disease course clusters in the Nordic JIA cohort are presented in Additional file 1: Table S1.

We also constructed an alternative definition of the outcome using five variables, the four described above in addition to the pain-VAS report at the 8-year follow-up. Both constructions corresponded reasonably well with the construction in the ReACCh-Out study, and the results of the external validation of the Canadian model were similar in the two cases. In both cases, we made a series of choices and essentially tuned the construction to obtain clusters that corresponded in relative size to those found in the Canadian study. We used linear dimensionality reduction and then the *K*-means or the *K*-medoids clustering algorithm [26] to construct clusters.

### Predictors in the Nordic and the Canadian model

The baseline predictors that we considered as candidates for the Nordic multivariable logistic regression model are previously published [19]. The following eight predictors constituted the final multivariable model: cumulative active joint count; erythrocyte sedimentation rate (ESR) mm/hour, measured as a continuous variable; C-reactive protein (CRP) mg/l, with values < 10 mg/l considered to be normal; morning stiffness > 15 min; physician’s global assessment of disease activity on a 10-cm VAS; presence of antinuclear antibodies (ANA) analyzed by immunofluorescence on Hep-2cells and tested at least twice with a minimum of 3 months apart; presence of human leucocyte antigen (HLA)-B27; and ankle joint arthritis. The first five variables were included a priori based on a clinical judgment and justified on the basis that these variables are central in the American College of Rheumatology (ACR) for clinical active disease [27].

The Canadian multivariable logistic regression model used 16 variables: active joint count, psoriatic arthritis, oligoarthritis, RF-negative polyarthritis, upper limb joint involvement, symmetric joint involvement, RF positivity, subtalar joint involvement, finger joint involvement, cervical spine involvement, ankle joint involvement, presence of morning stiffness, hip joint involvement, temporomandibular joint involvement, mid-foot involvement, and the presence of enthesitis. Details regarding measurement and assessment of these variables were previously reported [17].

### Model validation

This study presents external validation of the Canadian model and internal validation of the Nordic model. The Canadian model was tested for its ability to predict *severe disease course* and *non-achievement of remission* in a separate cohort from the one used to build the model. The Canadian model was first tested exactly as published by Guzman et al., and also after fine-tuning, i.e., with re-estimated coefficients. The Nordic model was tested for its ability to predict *severe disease course* by internal validation, involving repeated partitioning of the cohort in multiple training sets for model building and validation sets for model testing.

### Statistical analyses

Rypdal et al. constructed multivariable logistic regression models using a set of 5 pre-defined variables and a stepwise forward selection method to obtain additional variables from a set of 29 candidate variables. Variables with a *P* value > 0.05 were removed. Selections of variables were performed in training sets, and no more than 10 predictor variables were allowed in each of the models. The final model included 8 predictors, as previously described [19].

Guzman et al. constructed their model through a version of backwards elimination starting with a full model of 52 predictors and retaining 16 predictor variables in their multivariable logistic regression model. Both the Nordic and the Canadian models underwent internal validation with the repeated random split-sample technique and cross-validation in their respective cohorts.

#### External validation of the Canadian model

The model [17] is tested by computing the probability of *severe disease course* and *non-achievement of remission* according to the formula:

$$ p=\frac{1}{1+{e}^{-A}} $$

where *A* = *β*_0_ + *β*_1_*x*_1_ + … + *β*_16_*x*_16_ is a linear combination of predictors. Apart from the active joint count, all variables are dichotomous. In external validation, we used the coefficients *β*_*i*_ from the ReACCh-Out cohort exactly as published [17]. A probability of *severe disease course* and *non-achievement of remission* was computed for each patient in the Nordic cohort, and these probabilities were compared to the outcomes described above. By varying the probability threshold, pairs of corresponding sensitivity and specificity values were obtained, and consequently a receiver operating characteristic (ROC) curve. The area under the curve (AUC), or C-statistic, was computed from the ROC curve for each outcome. This is reported as the C-index. For each outcome, the uncertainty in the C-statistic was quantified by a standard bootstrapping (resampling) method and reported as interquartile range (IQR).

#### Testing of the Canadian model after fine-tuning

This involved re-estimating the coefficients *β*_*i*_ in subsets (training sets) of the Nordic cohort and evaluating the corresponding models (using the same method) as described above on disjoint validation sets. We used 500 repeated random splits into training and validation sets, and the median C-statistics with IQRs were computed. For each random split, we used 75% of available patients for training, and 25% for testing.

#### Internal validation of the Nordic model

The Nordic model was validated by constructing and training models on training sets and tested on disjoint validation sets as described above. For the Nordic model, the training involved not only the estimation of coefficients *β*_*i*_, but also the variable selection as reported [19]. The results for prediction of *non-achievement of remission* have been previously reported, but in the present study, we extended this analysis to prediction of *severe disease course*. For comparison, we also carried out this analysis for a univariate logistic regression model with cumulative active joint count at baseline as the only predictor. The sample size was determined by the number of patients with available data for analyses in the Nordic JIA cohort.

For the construction of the *severe disease course* outcome, there were 1 or more missing values for 248 of the 440 patients. Since *severe disease course* is a data-driven outcome, it was necessary to impute these missing values. For this purpose, we used the linear dimension-reduction algorithm in the Wolfram Mathematica software. The results presented in this study are without imputation of missing data for the predictor variables; thus, patients with 1 or more missing predictor variables were omitted from the testing of that particular model. For the external validation of the Canadian model, we lost 222 of 440 patients due to missing data in 1 or more of the 16 predictors. Most of these were missing tests of RF positivity (repeated twice at least 3 months apart). To test the effect of the missing predictor data on the main result, we performed a sensitivity analysis where we imputed missing data in predictor variables and re-tested the Canadian model. Results did not change significantly.

The statistical analyses in the current study were performed using Stata/MP version 15 and Wolfram Mathematica version 11.3.0.0.

## Results

Among the 440 patients in the Nordic cohort, 98/440 (22%) were identified with a severe disease course. This ratio is similar to the 125 (21%) of 609 patients identified with a severe disease course in the ReACCh-Out study. Altogether, 246/427 (58%) were not in remission off medication at the 8-year visit. The general characteristics of the 2 study populations are presented in Table 1, and detailed clinical characteristics of the groups of patients with severe disease course in the two cohorts are presented in Table 2.

**Table 2:** Baseline clinical characteristics for patients in the ReACCh-Out and the Nordic JIA cohort according to severe disease course or non-severe disease course

Characteristics	ReACCh-Out development cohort		Nordic validation cohort	
	Severe disease (*n* = 125)	Non-severe (*n* = 484)	Severe disease (*n* = 98)	Non-severe (*n* = 342)
Age at onset, years	9.9 (5.4–12.0), *n* = 123	6.9 (2.5–10.7), *n* = 474	8.1 (2.9–11.0)	5.2 (2.3–9.0)
Female, *n* (%)	88 (70.4)	325 (67.1)	78 (79.6)	213 (62.3)
Disease onset to diagnosis, months	5.6 (2.4–13.9)	3.3 (1.6–6.4)	2.4 (1.4–5.1), *n* = 94	1.4 (1.4–2.8), *n* = 321
Disease onset to enrollment, months	8.8 (4.9–17.0)	5.5 (2.8–9.9)	6.0 (6.0–9.0)	7.0 (6.0–8.0)
JIA category, *n* (%)				
Oligoarthritis	9 (7.2)	214 (44.2)	27 (27.6)	200 (58.5)
RF-neg. polyarthritis	44 (35.2)	85 (17.6)	37 (37.8)	57 (16.7)
RF-pos. polyarthritis	20 (16.0)	6 (1.2)	3 (3.1)	1 (0.3)
Systemic	10 (8.0)	37 (7.6)	2 (2.0)	15 (4.4)
Enthesitis-related	24 (19.2)	57 (11.8)	9 (9.2)	25 (7.3)
Psoriatic	4 (3.2)	32 (6.6)	1 (1.0)	5 (1.5)
Undifferentiated	14 (11.2)	53 (11.0)	19 (19.4)	39 (11.4)
Active joints, *n* (%)				
Cervical arthritis	21 (16.8)	8 (1.7)	22 (22.7)	16 (4.7)
Finger arthritis	86 (68.8)	122 (25.2)	63 (65.0)	72 (21.1)
Ankle arthritis	78 (62.4)	140 (28.9)	61 (62.9)	137 (40.1)
Hip arthritis	35 (28.0)	34 (7.0)	19 (19.6)	45 (13.2)
Cumulative active joint count^a^	13 (4–26)	2 (1–4)	9 (5–14)	2 (1–5)
Physician global assessment VAS	5.3 (3.2–7.2)	2.3 (1.0–4.6)	2.4 (1.0–4.7), *n* = 75	1.0 (0.3–2.1), *n* = 173
Parents’ global assessment VAS	3.6 (1.8–5.7), *n* = 114	1.3 (0.3–3.5), *n* = 440	2.3 (1.0–5.0), *n* = 76	0.9 (0.0–2.5), *n* = 195
Pain VAS	5.0 (2.7–6.8), *n* = 114	2.0 (0.5–5.0), *n* = 440	3.4 (1.1–5.0), *n* = 75	0.8 (0.0–2.8), *n* = 192
CHAQ	0.9 (0.3–1.4), *n* = 109	0.3 (0.0–0.8), *n* = 408	0.9 (0.3–1.4), *n* = 78	0.1 (0.0–0.7), *n* = 200
Morning stiffness, *n* (%)	102/124 (82.3)^b^	334/447 (74.7)^b^	60/86 (69.8)^c^	60/254 (23.6)^c^
ESR mm/hour	20 (9–45), *n* = 119	20 (9–36), *n* = 433	16 (8–39), *n* = 77	14 (8–25), *n* = 281
CRP mg/l	5.8 (0.4–34.0), *n* = 98	2.0 (0.1–10.0), *n* = 371	0.0 (0.0–22.5), *n* = 80	0.0 (0.0–10.0), *n* = 274
ANA positive, *n* (%)	54 (43.0)^d^	233 (48.0)^d^	22/95 (23.2)	93/332 (28.3)
RF positive, *n* (%)	24 (19.2)^d^	21 (4.3)^d^	4/70 (5.7)	6/171 (3.5)
HLA B27 positive, *n* (%)	18 (14.4)^d^	46 (9.5)^d^	23/96 (24.0)	63/314 (20.1)
Treatment by first study visit, *n* (%)				
NSAIDs	115/125 (92.0)	451/484 (93.2)	83/97 (85.6)	290/337 (86.1)
Joint injections	9/125 (7.2)	92/484 (19.0)	46/95 (48.4)	195/334 (58.4)
DMARDs	89/125 (71.2)	114/484 (23.6)	39/94 (41.5)	53/320 (16.6)
Biologics	2/125 (1.6)	0	0	0

Numbers are median interquartile range (IQR) unless otherwise specified

*VAS* visual analogue scale, *CHAQ* Childhood Health Assessment Questionnaire, *ESR* erythrocyte sedimentation rate, *CRP* C-reactive protein, *ANA* antinuclear antibodies, *RF* rheumatoid factor, *HLA B27* Human Leucocyte Antigen B27, *NSAID* non-steroidal anti-inflammatory drug, *DMARD* disease modifying antirheumatic drug

^a^The Nordic cohort used the cumulative joint count within 6 months of disease onset, and the ReACCh-Out cohort used the active joint count at baseline

^b^Morning stiffness > 30 min

^c^Morning stiffness > 15 min

^d^Values on ANA, RF, and HLA B7 for the Canadian cohort are after imputation

In the Nordic validation cohort, 66.2% were female. The baseline visit took place at a median of 7 (IQR 6–8) months after the first symptom of JIA, and the median time for assessment of the outcome was 98 (IQR 95–102) months after disease onset. Time from disease onset to JIA diagnosis was 1.6 (IQR 1.4–3.3) months. The median age at disease onset was 5.5 (IQR 2.5–9.7) years.

In the Canadian development cohort, 67.9% were female. The median time from disease onset to the baseline visit was 5.8 (IQR 3–11) months. The outcome was assessed on patients that attended at least six of eight planned visits, which correspond to a follow-up of 3 to 5 years. Time from first symptom to diagnosis was 3.7 (IQR 1.8–7.3) months, and the median age at disease onset was 8.4 (IQR 3.4–11.9) years.

### Model validation

**Fig. 1:**
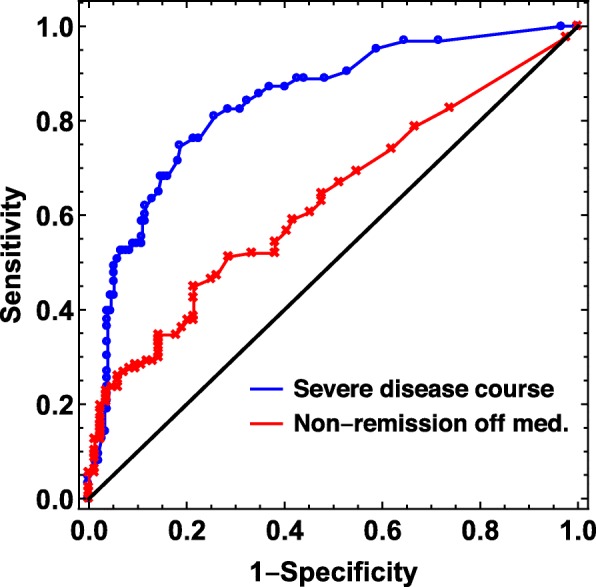
Receiver operating characteristic (ROC) curves showing external validation of the Canadian prediction model in the Nordic JIA cohort. Blue curve: using severe disease course as outcome. C-index with IQR = 0.85 (0.83–0.87). Red curve: using non-achievement of remission as outcome. C-index with IQR = 0.66 (0.63–0.68)

The external validation with *severe disease course* as outcome resulted in a C-index of 0.85, and bootstrapping gave an estimated IQR of 0.83–0.87. For *non-achievement of remission*, the C-index was 0.66 (IQR 0.63–0.68) (Table 3). The corresponding ROC curves for the external validation are shown in Fig. 1, and the calibration plots are shown in the Additional file 2: Figure S1. The alternative construction of *severe disease course*, based on five rather than four variables at the 8-year follow-up, gave a C-index of 0.84 with an IQR of 0.82–0.87. After imputation of missing data in predictor variables the C-index was 0.83.

**Table 3:** C-indices for testing of Canadian and Nordic prediction models

Prediction model	Severe disease course outcome	Non-achievement of remission outcome	Validation method
Original Canadian model	0.85 (0.83–0.87)	0.66 (0.63–0.68)	External validation (bootstrapping)
Canadian model fine-tuned for Nordic population	0.85 (0.81–0.89)	0.69 (0.65–0.73)	Fine-tuning (repeated random splits)
Nordic model	0.90 (0.86–0.92)	0.78 (0.72–0.82)^a^	Internal validation (repeated random splits)

C-indices with median interquartile range (IQR)

C-index presented includes patients with systemic JIA, except for ^a^the C-index for the Nordic model and the outcome non-achievement of remission previously published by Rypdal et al. [19]

**Fig. 2:**
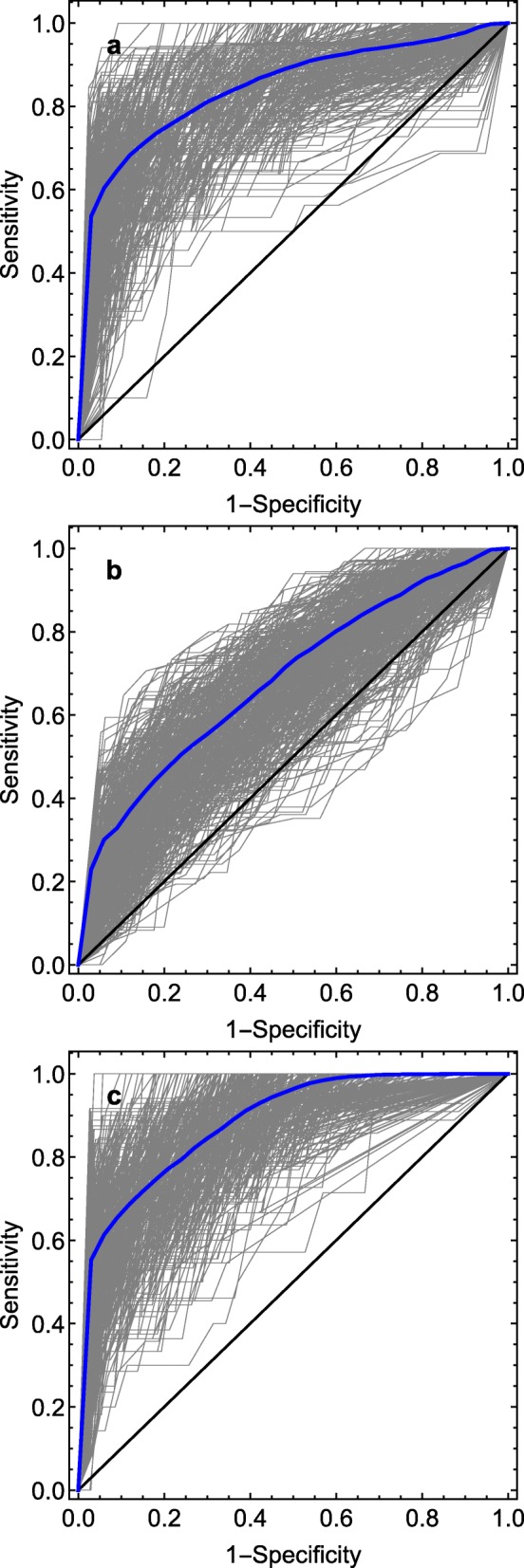
Receiver operating characteristic (ROC) curves showing results of fine-tuned models in the Nordic JIA cohort for different outcomes. **a** Fine-tuned Canadian prediction model using severe disease course as outcome. **b** Fine-tuned Canadian prediction model using non-achievement of remission as outcome. **c** Internal validation of Nordic prediction model using severe disease course as outcome

After fine-tuning in training sets, the Canadian model had a median C-index of 0.85 (IQR 0.80–0.89) with *severe disease course* as outcome (Table 3 and Fig. 2a). The same analysis with *non-achievement of remission* as outcome gave a C-index of 0.69 (IQR 0.65–0.73) (Table 3, Fig. 2b). The model variables and their corresponding *β*_*i*_-coefficients for the original ReACCh-Out model and the model fine-tuned to the Nordic population are presented in Table 4.

**Table 4:** Canadian prediction model with respective *β*_*i*_ coefficients before and after fine-tuning in the Nordic JIA cohort

Predictor variables in the Canadian model	Original ReACCh-Out cohort^a^	Fine-tuned in the Nordic cohort^b^
Constant	Intercept = − 2.92	Intercept = 2.76
Active joint count, *n* = 440	0.18	0.21
Psoriatic arthritis, *n* = 440	− 1.23	− 1.40
Oligoarthritis, *n* = 440	− 1.14	− 0.72
RF-negative polyarthritis, *n* = 440	− 0.49	− 0.68
Upper limb joint involvement, *n* = 440	0.75	− 1.11
Symmetric joint involvement, *n* = 439	− 0.88	0.68
RF positivity, *n* = 241	1.31	− 1.06
Subtalar joint involvement, *n* = 439	− 1.42	− 2.81
Finger joint involvement, *n* = 439	− 0.31	1.31
Cervical spine involvement, *n* = 439	0.84	0.38
Ankle joint involvement, *n* = 439	0.48	− 0.25
Presence of morning stiffness, *n* = 340	0.56	1.64
Hip involvement, *n* = 439	0.06	− 0.50
TMJ-involvement, *n* = 439	1.50	0.09
Mid foot involvement, *n* = 439	0.54	0.39
Presence of enthesitis, *n* = 437	0.86	1.26

^a^Coefficients found by logistic regression in the Canadian cohort, previously reported [17]

^b^The changes in coefficients after fine-tuning in the Nordic JIA cohort

We also performed *internal validation* of our Nordic model using *severe disease course* as outcome. This gave a median C-index of 0.90 (IQR 0.86–0.92) (Table 3, Fig. 2c). Ultimately, we tested a very simple prediction model with cumulative active joint count at baseline as the only predictor. For this model, a C-index of 0.85 (IQR 0.82–0.88) was estimated. The corresponding ROC curve is presented in Additional file 3: Figure S2.

## Discussion

A clinically useful prediction model for long-term outcome in JIA should be tested for reliability and accuracy across cohorts, countries, and ethnicities to avoid overestimating the predictive performance of the model. To our knowledge, the two studies presented in this issue are the first where prediction models for unfavorable outcomes in JIA are tested on cohorts completely different to those used to construct the models.

The main result of this study is that the external validation of the Canadian prediction model yielded excellent predictive performance with a C-index of 0.85 (IQR 0.83–0.87) for *severe disease course* in the Nordic cohort. The result is consistent with the internal validation in the Canadian cohort, where a C-index of 0.85 was obtained [17]. The Canadian model was also tested after fine-tuning on repeated random splits, giving a similar result to the ones in external validation. Internal validation of the Nordic model also indicated excellent performance (C-index of 0.90) for predicting a *severe disease course.*

In all comparisons, C-indices for prediction of *severe disease course* were higher than for prediction of *non-achievement of remission*.

Recently, several prediction models in JIA have been published, but predictive abilities are suboptimal, and none of them have been externally validated in an entirely different population [14–16, 18, 19]. The current study highlights two key points: (1) The choice of outcome to be predicted is essential for predictive performance and perhaps more important than model design. (2) Prediction models based on a few key variables may have similar predictive ability to more complex models, at least for the outcomes examined in this study.

The first point is supported by the comparison of *non-achievement of remission* and *severe disease course.* It seems the latter defines a narrower and more homogeneous group of patients that is easier to identify and predict. In our opinion, severe disease course is clinically relevant because it captures a group of JIA patients most severely affected by the disease. This adverse outcome may correspond better with the threshold in many countries for initiating biologic treatment and therefore be a better prediction target to guide early aggressive treatment [8–10, 28].

The second point is supported by observing that in this study, the predictive abilities of the most complex models are not much better than those of simpler models. The Nordic model for prediction of *non-achievement of remission* was designed with specific conditions in place to ensure model simplicity. It is comparable to the Canadian model in performance. However, the Canadian model is based on 16 variables and may be more difficult to use in clinical practice, even though an available online calculator is easy to use. Besides its predictive performance, one of the key features of a good clinical prediction rule is simplicity [29].

To further investigate the potential of very simple prediction models, we also assessed a univariate logistic regression model using cumulative active joint count during the first 6 months after disease onset as the only predictor. The model achieved high predictive performance for *severe disease course*, and we take this as an indication that model simplification is feasible. However, the high predictive ability of this very simple model may be explained by the dependence between cumulative active joint count at baseline and the cumulative active joint count later in the disease.

Simple prediction models may perform well for a large group of JIA patients, where the total number of joints affected explains much of the disease burden, but they may be of little use for patients with, for example, systemic JIA or enthesitis-related arthritis, where the severity of the disease may be strongly associated with other clinical features [30]. The heterogeneity of JIA is therefore an argument against oversimplified prediction models, and multivariable models may have greater applicability across the whole spectrum of JIA. While separate models for different JIA categories may be more accurate [15], they may add complexity to prediction.

### Study strengths and limitations

The main strength of this work is that we validate a model constructed in the Canadian cohort in the completely separate Nordic cohort. Both studies were multicenter, prospective, longitudinal studies and collected extensive clinical information. However, both the Canadian and Nordic models were constructed starting from a large number of clinical variables, which may have increased the risk of retaining uninformative predictors in the models and overfitting. A weakness of our study is missing data in predictor and outcome variables, which is a common problem in prediction studies [31]. We have tried to address this issue by imputing the values for the variables used in the data-driven outcome and by not omitting patients who lack information on predictor variables. Selecting only patients with complete data may lead to biased results.

In conclusion, we found excellent predictive performance of both the Canadian and Nordic prediction models for predicting a *severe disease course* in children with JIA. Severe disease course was identified using an implicit, data-driven clustering method. Identifying an objective definition of a severe disease course was beyond the scope of this paper, but a clinical definition of severe disease course in JIA is clearly needed. Future studies on prediction models in JIA are necessary, focusing not only on constructing simplified prediction models, but also on determining improved disease-outcome definitions in JIA. Once objective outcome definitions are in place, we can use the knowledge gained from the Nordic-Canadian collaboration to develop new models that can be tested in a third and independent cohort. The ultimate step will be testing the model in a randomized controlled trial to verify if it can significantly improve patient outcomes. The aim is to develop models that can be used in every day clinical practice. We have developed a smartphone application for the Nordic model, and online web-based calculators exist for both the Nordic (http://predictions.no) and the Canadian (https://shiny.rcg.sfu.ca/jia-sdcc/) models [17, 19]. These tools can easily be extended to new models. As we better understand the accuracies and limitations of the models, physicians may incorporate them in their overall assessments to improve outcome in JIA.

## Supplementary information

**Additional file 1: Table S1.** Characteristics of the four clusters identified in the Nordic JIA cohort. Cluster 3 and 4 correspond to the severe disease course outcome defined in the ReACCh-Out cohort.

**Additional file 2: Figure S1.** Calibration curves for the Canadian model in the Nordic JIA cohort. Each point represents one tenth of the patient sample, arranged from lowest to highest probability of the outcome. A: For predicting severe disease course. B: For predicting non-achievement of remission.

**Additional file 3: Figure S2.** Receiver operating characteristic (ROC) curve showing the result of the univariate logistic regression model with cumulative active joint count as the predictor variable and severe disease course as the outcome. C-index of 0.85 (IQR 0.82–0.88).

## Data Availability

The datasets generated and/or analyzed during the current study are not publicly available for ethical and privacy reasons but are available from the Nordic Study group of Pediatric Rheumatology (NoSPeR) on reasonable request.

## Abbreviations

JIA: Juvenile idiopathic arthritis
IQR: Interquartile range, 25th, 75th centiles
ReACCh-Out: Research in Arthritis in Canadian Children Emphasizing Outcomes
TRIPOD: Transparent reporting of a multivariable prediction model for individual prognosis or diagnosis
VAS: Visual analogue scale
CHAQ: Childhood Health Assessment Questionnaire
PhS: Physical Summary Score
CHQ-PF50: Child Health Questionnaire Parent form
ANA: Antinuclear antibodies
RF: Rheumatoid factor
CRP: C-reactive protein
ESR: Erythrocyte sedimentation rate
ACR: American College of Rheumatology
HLA-B27: Human leucocyte antigen B27
ROC: Receiver operating characteristic
AUC: Area under the (ROC) curve
NSAIDs: Non-steroidal anti-inflammatory drugs
DMARDs: Disease modifying antirheumatic drugs
