# Supplementary material for: Validation of prediction models of severe disease course and non-achievement of remission in juvenile idiopathic arthritis: part 1—results of the Canadian model in the Nordic cohort
Source: Arthritis Res Ther. 2019 Dec 5;21:270. doi: 10.1186/s13075-019-2060-2 (PMC6896283; doi:10.1186/s13075-019-2060-2)
Supplement: Supplementary file 1 — Additional file 1: Table S1. Characteristics of the four clusters identified in the Nordic JIA cohort. Cluster 3 and 4 correspond to the severe disease course outcome defined in the ReACCh-Out cohort. [file 13075_2019_2060_MOESM1_ESM.docx]

| **Additional file 1: Table S1.** Characteristics of the four clusters identified in the Nordic JIA cohort, cluster 3 and 4 correspond to the severe disease course outcome defined in the ReACCh-Out cohort | | | | |
| --- | --- | --- | --- | --- |
| **Characteristics** | **Cluster 1** | **Cluster 2** | **Cluster 3** | **Cluster 4** |
| Age at onset, years | 5.4 (2.3-9.5)  n=212 | 4.9 (2.3-8.5)  n=130 | 7.5 (2.8-10.9)  n=75 | 9.5 (1.9-11.7)  n=23 |
| Female, n (%) | 130/212 (61.3) | 83/130 (63.9) | 57/75 (76.0) | 21/23 (91.3) |
| Time onset to diagnosis, months | 1.4 (0.4-2.7)  n=199 | 1.1 (0.4-3.0) n=122 | 2.3 (0.6-5.1)  n=71 | 2.6 (1.3-5.7)  n=23 |
| Time onset to enrollment, months | 7 (6-8) n=212 | 7 (6-8)  n=130 | 6 (6-8)  n=75 | 6 (6-12)  n=23 |
| **JIA category, n (%)** |  |  |  |  |
| Oligoarthritis | 128/212 (60.4) | 72/130 (55.4) | 21/75 (28.0) | 6/23 (26.1) |
| RF-neg. polyarthritis | 31/212 (14.6) | 26/130 (20.0) | 24/75 (32.0) | 13/23 (56.5) |
| RF-pos. polyarthritis | 1/212 (0.5) | 0 | 2/75 (2.7) | 1/23 (4.4) |
| Systemic | 13/212 (6.1) | 2/130 (1.5) | 2/75 (2.7) | 0 |
| Enthesitis-related | 12/212 (5.7) | 13/130 (10.0) | 9/75 (12.0) | 0 |
| Psoriatic | 5/212 (2.4) | 0 | 1/75 (1.3) | 0 |
| Undifferentiated | 22/212 (10.4) | 17/130 (13.1) | 16/75 (21.3) | 3/23 (13.0) |
| **Active joints, n (%)** |  |  |  |  |
| Cervical arthritis | 11/212 (5.2) | 5/130 (3.9) | 15/74 (20.3) | 7/23 (30.4) |
| Finger arthritis | 43/212 (20.3) | 29/130 (22.3) | 46/74 (62.2) | 17/23 (73.9) |
| Ankle arthritis | 79/212 (37.3) | 58/130 (44.6) | 44/74 (59.5) | 17/23 (73.9) |
| Hip arthritis | 27/212 (12.7) | 18/130 (13.9) | 15/74 (20.3) | 4/23 (17.4) |
| Cumulative active joint count | 2 (1-4)  n=212 | 2 (2-5) n=130 | 7 (4-13) n=75 | 15 (8-27) n=23 |
| Physician global assessment VAS | 1.0 (0.0-1.7) n=107 | 1.3 (0.7-3.0) n=66 | 2.3 (0.7-4.7) n=58 | 2.3 (2.0-5.7) n=17 |
| Parents global assessment VAS | 0.7 (0.0-2.0) n=119 | 1.0 (0.3-3.0) n=76 | 2.7 (1.0-5.0) n=58 | 2.0 (1.0-5.0) n=17 |
| Pain VAS | 0.3 (0.0-2.7) n=119 | 0.3 (0.0-2.7) n=73 | 3.3 (1.3-5.0) n=59 | 3.7 (1.0-5.3) n=16 |
| CHAQ | 0.1 (0.0-0.6) n=122 | - 1. (0.0-1.0)   n=78 | 0.9 (0.3-1.4)  n=61 | 0.9 (0.5-1.4)  n=17 |
| Morning stiffness >15 min., n (%) | 29/160 (18.1) | 31/94 (33.0) | 47/67 (70.2) | 13/19 (68.4) |
| ESR mm/hour | 12.5 (7-22)  n=170 | 15 (10-30)  n=111 | 15.5 (7.5-40.5)  n=56 | 16 (10-39)  n=21 |
| CRP > 10 mg/ml | 28/160 (17.5) | 31/114 (27.2) | 16/57 (28.7) | 9/23 (39.1) |
| ANA positive, n (%) | 45/204 (22.1) | 49/128 (38.3) | 20/73 (27.4) | 2/22 (9.1) |
| RF positive, n (%) | 5/105 (4.8) | 1/66 (1.5) | 3/55 (5.5) | 1/15 (6.7) |
| HLA B27 positive, n (%) | 29/190 (15.3) | 34/124 (27.4) | 17/73 (23.3) | 6/23 (26.1) |
| **Treatment by first study visit, n (%)** |  |  |  |  |
| NSAIDs | 174/208 (83.7) | 116/129 (89.9) | 63/74 (85.1) | 20/23 (87.0) |
| Joint injections | 112/206 (54.4) | 83/128 (64.8) | 36/72 (50.0) | 10/23 (43.5) |
| DMARDs | 23/200 (11.5) | 30/120 (25.0) | 31/72 (43.1) | 8/22 (36.4) |
| Biologics | 0 | 0 | 0 | 0 |
| Numbers are median interquartile range (IQR) unless otherwise specified. VAS; Visual analogue scale, CHAQ; Childhood Health Assessment Questionnaire, ESR; Erythrocyte sedimentation rate, CRP; C-reactive protein, ANA; Antinuclear antibodies, RF; Rheumatoid factor, HLA B27; Human Leucocyte Antigen B27, NSAID; Non-steroidal anti-inflammatory drug, DMARD; Disease modifying antirheumatic drug. | | | | |
