# Supplementary material for: Validation of prediction models of severe disease course and non-achievement of remission in juvenile idiopathic arthritis: part 1—results of the Canadian model in the Nordic cohort
Source: Arthritis Res Ther. 2019 Dec 5;21:270. doi: 10.1186/s13075-019-2060-2 (PMC6896283; doi:10.1186/s13075-019-2060-2)
Supplement: Supplementary file 2 — Additional file 2: Figure S1. Calibration curves for the Canadian model in the Nordic JIA cohort. Each point represents one tenth of the patient sample, arranged from lowest to highest probability of the outcome. A: For predicting severe disease course. B: For predicting non-achievement of remission. [file 13075_2019_2060_MOESM2_ESM.docx]

**Additional file 2: Figure S1.** Calibration curves for the Canadian model in the Nordic JIA cohort. Each point represents one tenth of the testing patient sample, arranged from lowest to highest probability of the outcome.

A: For predicting severe disease course.

B: For predicting non-achievement of remission.
