# Supplementary material for: Validation of prediction models of severe disease course and non-achievement of remission in juvenile idiopathic arthritis: part 1—results of the Canadian model in the Nordic cohort
Source: Arthritis Res Ther. 2019 Dec 5;21:270. doi: 10.1186/s13075-019-2060-2 (PMC6896283; doi:10.1186/s13075-019-2060-2)
Supplement: Supplementary file 3 — Additional file 3: Figure S2. Receiver operating characteristic (ROC) curve showing the result of the univariate logistic regression model with cumulative active joint count as the predictor variable and severe disease course as the outcome. C-index of 0.85 (IQR 0.82–0.88). [file 13075_2019_2060_MOESM3_ESM.docx]

**Additional file 3: Figure S2.** Receiver operating characteristic (ROC) curve showing the result of the univariate logistic regression model with cumulative active joint count as the predictor variable and severe disease course as the outcome. C-index of 0.85 (IQR 0.82-0.88).
